# HLA-DRB1 shared epitope genotyping using the revised classification and its association with circulating autoantibodies, acute phase reactants, cytokines and clinical indices of disease activity in a cohort of South African rheumatoid arthritis patients

**DOI:** 10.1186/ar3479

**Published:** 2011-10-06

**Authors:** Pieter WA Meyer, Bridget Hodkinson, Mahmood Ally, Eustasius Musenge, Ahmed A Wadee, Heidi Fickl, Mohammed Tikly, Ronald Anderson

**Affiliations:** 1 Medical Research Council Unit for Inflammation and Immunity, Department of Immunology, Faculty of Health Sciences, University of Pretoria, and Tshwane Academic Division of the National Health Laboratory Service, Bophelo Road, Pretoria, 0001, South Africa; 2 Division of Rheumatology, Chris Hani Baragwanath Hospital, Faculty of Health Sciences, University of the Witwatersrand, Chris Hani Road, Johannesburg, 2013, South Africa; 3 Department Internal Medicine, Faculty of Health Sciences, University of Pretoria, Bophelo Road, Pretoria, 0001, South Africa; 4 Epidemiology and Biostatistics Division, School of Public Health, Faculty of Health Sciences, University of the Witwatersrand, York Road, Johannesburg, 2193, South Africa; 5 Department of Immunology, School of Pathology, University of the Witwatersrand and the National Health Laboratory Service, Hospital Street, Braamfontein, 2001, Johannesburg, South Africa

**Keywords:** anticyclic citrullinated peptide antibodies, C-reactive protein, fibroblast cytokines, macrophage cytokines, rheumatoid factor, serum amyloid A, Th1/Th2 cytokines

## Abstract

**Introduction:**

The revised shared epitope (SE) concept in rheumatoid arthritis (RA) is based on the presence (S) or absence (X) of the SE RAA amino acid motif at positions 72 to 74 of the third hypervariable region of the various human leucocyte antigen (HLA)-DRB1 alleles. The purpose of this study was to investigate SE subtypes on the basis of the American College of Rheumatology 1987 revised criteria for the classification of RA in a cohort of South African RA patients (*n *= 143) and their association with clinical and circulating biomarkers of disease activity (autoantibodies, acute phase reactants and cytokines).

**Methods:**

Genomic DNA was analysed using high-resolution recombinant sequence-specific oligonucleotide PCR typing of the HLA-DRB1 allele. Subtypes of the SE were classified according to the amino acids at positions 72 to 74 for the RAA sequence, and further sub-divided according to the amino acids at positions 70 and 71, which either contribute to (S2, S3P), or negate (S1, S3D) RA susceptibility. Disease activity was assessed on the basis of (1) Disease Activity Score in 28 joints using C-reactive protein (CRP), (2) rheumatoid factor (RF), (3) CRP and (4) serum amyloid A by nephelometry, anticyclic citrullinated peptide antibodies (aCCP) by an immunofluorometric procedure, and cytokines by multiplex bead array technology.

**Results:**

Of the 143 RA patients, 81 (57%) were homozygous (SS) and 50 (35%) were heterozygous (SX) for the SE alleles with significant overexpression of S2 and S3P (respective odds ratios (ORs) 5.3 and 5.8; *P *< 0.0001), and 12 (8%) were classified as no SE allele (XX). Both the SS and SX groups showed a strong association with aCCP positivity (OR = 10.2 and *P *= 0.0010, OR = 9.2 and *P *= 0.0028, respectively) relative to the XX group. Clinical scores and concentrations of the other biomarkers of disease activity (RF, CRP and T helper cell type 1 (Th1), Th2, macrophage and fibroblast cytokines) were also generally higher in the SS group than in the SX and XX groups.

**Conclusions:**

RA susceptibility alleles investigated according to revised criteria for the classification of RA were significantly increased in South African RA patients and strongly associated with aCCP in particular as well as with circulating cytokines and disease severity.

## Introduction

Rheumatoid arthritis (RA) is a debilitating autoimmune disease that has no clearly defined aetiology, although there is a definite genetic predisposition and associated risk factors [1]. The shared epitope (SE) concept in relation to genetic predisposition was first described in 1986 and has evolved from the classic HLA-DRB1*01, HLA-DRB1*04 and HLA-DRB1*10 associations [2-4] to the identification of the RAA amino acid motif at positions 72 to 74 of the third hypervariable region of the different human leucocyte antigen (HLA)-DRB1 chains as the definitive SE [3-5]. This concept has been extended by Gao *et al*. [6] to include the amino acid residues at positions 71 and 76 and, recently, to a new classification which incorporates the modulatory activities of the amino acids at positions 70 and 71 in addition to the RAA motif at positions 72 to 74 [6-8].

Although the primary triggering autoantigens in RA have not been described to date, it is noteworthy that associations between the various HLA-DRB1 SE subtypes with disease susceptibility and/or severity and the presence of circulating anticitrullinated peptide antibodies have been described [9-18]. In addition, HLA-DRB1 SE genotyping and measurement of anticyclic citrullinated peptide antibodies (aCCP) and, to a lesser extent, rheumatoid factor (RF) have the potential to predict future development of RA [10,14,17,19-21]. Taken together, these associations between HLA-DRB1 SE genotype, aCCP and disease susceptibility and/or severity appear to be compatible with the presentation of citrullinated autoantigens by HLA-DRB1 SE subtypes as an immunopathogenic mechanism in RA.

While lacking diagnostic specificity, the measurement of circulating cytokines and chemokines and acute phase reactants, combined with the detection of aCCP and RF, has the potential to predict the time until onset of clinical disease [22,23] as well as disease severity [24-27]. Nonetheless, in relatively few studies have researchers undertaken a composite analysis of SE genotyping and measurement of circulating aCCP, cytokines, chemokines and acute phase reactants as a strategy not only to identify interactions between these alleles and biomarkers but also to establish which combinations of these are most strongly associated with disease severity. These issues were addressed in the current study of a cohort of predominantly African patients with RA of two years' duration or less. To our knowledge, this is the first study to measure the frequency of the various SE subtypes according to the du Montcel classification system [7] in this patient population.

## Materials and methods

Following approval by the Research Ethics Committees of the Faculties of Health Sciences of the University of Pretoria and University of the Witwatersrand, 143 patients who presented to the rheumatology clinics of two tertiary hospitals in the Gauteng Province of South Africa (Chris Hani Baragwanath Hospital, Soweto, and Steve Biko Academic Hospital, Pretoria) were recruited to participate in the study. Prior informed consent was obtained from all participants, all of whom met the 1987 American College of Rheumatology criteria for RA [28]. All of the patients were disease-modifying antirheumatic drug (DMARD)-naïve and HIV-seronegative and had RA symptoms of two years' duration or less. The Disease Activity Score in 28 joints (DAS28) on the basis of CRP level (DAS28-CRP) was used to assess disease activity upon presentation [29,30]. "Erosive disease" was defined as the presence of marginal joint erosions on standard X-rays of the hands and feet. These clinical assessments were performed by two experienced rheumatologists (BH and MA). Demographic data for this cohort are shown in Table 1.

**Table 1:** Demographic, laboratory and clinical data for the RA patient group (*n *= 143)

Parameters	Data	Median [IQR]	Range
Age (years)	48 ± 12	48 [41 to 56]	20 to 75
Females, *n *(%)	118 (82.5%)		
Blacks, *n *(%)	124 (86.7%)		
DAS28-CRP	5.6 ± 1.3	5.7 [4.8 to 6.7]	2.3 to 8.7
Disease duration (months)	12 ± 7	10 [6 to 18]	0 to 25
Larsen radiographic scores	23 ± 14	19 [14 to 28]	1 to 66
RF (IU/ml)	490 ± 795	168 [38 to 595]	4 to 5,350
aCCP (U/ml)	669 ± 636	492 [85 to 1,099]	2 to 2,527
CRP (μg/L)	24 ± 32	14 [5 to 32]	0 to 198
SAA (μg/ml)	62 ± 123	14 [5 to 65]	1 to 882

aCCP = anticyclic citrullinated peptide antibodies; CRP = C-reactive protein; DAS28 = Disease Activity Score in 28 joints; RF = rheumatoid factor; SAA = serum amyloid A. Data are means ± SD, n (%), medians [25th to 75th IQR] or raw data as indicated.

### Autoantibodies and acute phase reactants

Venous blood (30 ml) was collected in commercial sample test tubes containing a gel separator. The blood samples were kept at room temperature to clot and then spun at 3,000 rpm for 10 minutes), followed by collection of the serum, aliquoting and storage at -20°C until needed for analysis.

RF (composite immunoglobulin M (IgM), IgG and IgA), CRP and serum amyloid A (SAA) were assayed by nephelometry (BN ProSpec System; Siemens Healthcare Diagnostics, Deerfield, IL, USA). As per the manufacturer's supplier controls, RF, CRP and SAA results were considered positive when their individual concentrations exceeded 11 IU/ml, 5 μg/ml and 6.8 μg/ml, respectively. aCCP were measured using a second-generation immunofluorometric procedure using the ImmunoCAP Phadia 250 assay system with reagents and controls provided by the manufacturer (Phadia AB, Uppsala, Sweden). A concentration greater than 10 U/ml was deemed positive.

### Serum cytokines, chemokines and growth factors

Serum cytokines, chemokines and growth factors were measured using the Bio-Plex multiplex suspension array system with xMAP technology (Bio-Rad Laboratories, Hercules, CA, USA), which enables simultaneous detection and quantitation of multiple different analytes in a single sample. The system uses an array of microspheres in liquid suspension that are conjugated with a mAb specific for a target protein. A wide range of standards (0.38 to 91,756.00 pg/ml) was used to enable quantitation of the individual cytokines by using a Bio-Plex array reader (Bio-Rad Laboratories) with a dual laser detector and real-time digital signal processing. The following analytes were measured: IL-1β, IL-1Ra, IL-2, IL-4, IL-6, IL-7, IL-8, IL-10, IL-12, IL-17, IFN-γ, TNF, granulocyte colony-stimulating factor (G-CSF), granulocyte macrophage colony-stimulating factor (GM-CSF), chemokine (C-C motif) ligand 2 (CCL2), CCL4 and vascular endothelial growth factor (VEGF). The upper limits of normal for these analytes were calculated as the mean +2 SD for 10 healthy control subjects (five female and five male, average age 44.4 ± 15.0 years, age range 26 to 65 years).

### Typing of human leucocyte antigen-DRB1 alleles

Genomic DNA was obtained by using the Maxwell 16 System with the Maxwell 16 Blood DNA Purification Kit (Promega, Madison, WI, USA) to extract DNA from whole blood, which was collected in ethylenediaminetetraacetic acid (EDTA) sample tubes. The patients' HLA-DRB1 alleles were determined by using a DNA-based high-resolution typing method, the LABType HD Class II DRB1 Typing Test (One Lambda Inc, Canoga Park, CA, USA), with reverse sequence-specific oligonucleotide probes and Luminex technology. The target DNA (*HLA-DRB1 *gene) was amplified by PCR using group-specific primers biotinylated for detection with streptavidin R-phycoerythrin conjugate. The PCR product was then denatured and hybridised to complementary DNA probes conjugated to fluorophores. Bound DNA was detected on the Luminex system, and software (HLA Fusion&#8482 Software Version 2.0, One Lambda, Inc.,21001 Kittridge Street, Canoga Park, CA, USA)was used to map the reaction patterns to those associated with published *HLA *gene sequences and assign the represented HLA-DRB1 alleles. HLA alleles were assigned according to the RAA amino acid sequence at positions 72-74 as SE (S) and non-SE (X), and the S group was further subdivided according to amino acid groups at positions 70 and 71 as described by du Montcel *et al*. [7] (see Table 2). The International Immunogenetics Information System database [31,32] and the Allele*Frequencies in Worldwide Populations Net Database [33] were used to establish regional and ethnicity-matched HLA-DRB1 frequencies in sub-Saharan Africa. The non-SE patients with the no SE allele (XX) genotype (*n *= 12) were used as controls in the calculation of contingency tables.

**Table 2:** Shared epitope classification according to amino acid sequence at positions 70-74

Allele classification	Amino acid sequence positions 70 to 74	HLA-DRB1 alleles
S1	D-E-RAA	*01:03, *04:02, *11:02-:03, *13:01-:02, *13:04, *13:36, *13:40
	Q-A-RAA	*15
S2	Q-K-RAA	*04:01, *04:09, *04:13, *04:35, *04:66
	D-K-RAA	*13:03
S3D	D-R-RAA	*11:01, *11:04, *11:27, *12, *13:05-:06, *13:25, *14:22, *16
S3P	Q-R-RAA	*01:01-:02, *04:04-:05, *04:08, *04:10
	R-R-RAA	*10:01
X	Q-K-RGR	*03
	Q-R-RAE	*04:03, *04:07, *04:11
	D-R-RGQ	*07
	D-R-RAL	*08
	R-R-RAE	*09:01, *14:01, *14:04

HLA = human leucocyte antigen. Data in right column from Gao *et al*. [6].

### Statistical methods

Descriptive and inferential statistics techniques were used in the analyses. Tests for association of contingency tables were performed using either the two-tailed Fisher's exact test or the χ^2 ^test with the Yates correction. One-way analysis of variance (ANOVA) was performed using the Kruskal-Wallis test for nonparametric data for more than two groups, or the Mann-Whitney *U *test when two groups were compared. Logistic regression was used for binary outcome variables, and the OR and corresponding 95% confidence interval were reported. *P *< 0.05 was considered statistically significant. The analyses were done using Stata software (Stata Corp, College Station, TX, USA).

## Results

### Human leucocyte antigen-DRB1 incidence

All of the "classical" RA-associated HLA-DRB1*01, HLA-DRB1*04 and HLA-DRB1*10 alleles showed statistically significant ORs, with HLA-DRB1*04 the most prominent (Table 3), underscoring the significance of the HLA-DRB1 SE previously reported in the African population [34-38]. The HLA-DRB1 alleles were categorized according to the new classification as being homozygous for the SE allele (SS), heterozygous for the SE allele (SX) or XX. The majority (92%) of the patients had at least one allele associated with the amino acid motif. More than half (57%) of the patients typed were homozygous (SS), 35% were heterozygous (SX) and only 8% had no associated HLA-DRB1 allele (XX). The SS, SX and XX allele distribution among the African subgroup of the cohort (*n *= 124) was 58% (*n *= 72), 35% (*n *= 43) and 7% (*n *= 9), respectively. The other South African population groups (Caucasian, Asian and mixed ancestry) were not meaningfully represented, however.

**Table 3:** Human leucocyte antigen-DRB1 rheumatoid arthritis-associated alleles, odds ratios and confidence intervals in the rheumatoid arthritis patient cohort

Risk allele	RA patients, *n *(%) (*n *= 143)	Controls*, *n *(%) (*n *= 1,104)	OR (95% CI)	*P *value
HLA-DRB1*01	26 (18.2)	93 (8.4)	2.4 (1.5 to 3.9)	0.0007
HLA-DRB1*04	61 (42.7)	41 (3.7)	19.3 (12.2 to 30.4)	<0.0001
HLA-DRB1*10	10 (7.0)	36 (3.3)	2.2 (1.0 to 4.6)	0.0338

HLA = human leucocyte antigen; OR = odds ratio; RA = rheumatoid arthritis. *Data from Robinson and colleagues [31,32].

Classification of RA-associated HLA-DRB1 alleles according to the du Montcel *et al*. classification system [7] revealed that S2 and S3P had significantly higher allele frequencies than those found in the general sub-Saharan population [32,33]. S3D and the non-epitope-associated alleles (X) were not significantly different in the RA groups compared to the general sub-Saharan population. As shown in Table 4, S1 was significantly undertransmitted in our population.

**Table 4:** Comparison of allele carrier frequencies in RA cases and controls in accordance with the du Montcel classification

Allele	RA patients, *n *(%) (*n *= 143)	Controls^*^, *n *(%) (*n *= 1,104)	**Χ**^**2 **^**test**	OR (95% CI)	*P *value
S1	55 (39)	853 (77)	40.3	0.4 (0.3 to 0.5)	<0.0001
S2	28 (20)	110 (10)	10.3	2.0 (1.3 to 3.2)	0.0013
S3D	38 (27)	361 (33)	0.1	0.9 (0.6 to 1.3)	0.7060
S3P	61 (43)	313 (28)	9.6	1.6 (1.2 to 2.2)	0.0019
X	64 (45)	616 (56)	3.6	0.8 (0.6 to 1.0)	0.0571

RA = rheumatoid arthritis. du Montcel classification is from du Montcel *et al*. [7]. *Data from Robinson and colleagues [31,32].

### Relationship of shared epitope classification and autoantibodies

Analysis (by one-way ANOVA and Mann-Whitney *U *test) of the median values of autoantibodies revealed that they were generally higher in the SS group than in the SX group. Significant associations were noted for aCCP (SS vs SX vs XX; *P *= 0.0298) and for RF (SS vs XX, *P *= 0.0129; SX vs XX, *P *= 0.0273).

As X alleles have been shown to be undertransmitted in the RA cohort (that is, noncarriers of risk alleles) (see Table 4), they were used as the control group within the RA cohort to determine the possible associations of the HLA-DRB1 genotype with RA-associated autoantibodies by comparing the total positive and negative results for the RA-associated antibodies (RF and aCCP) in the risk groups with those for the control group (XX). As shown in Table 5, both RF and aCCP seropositivity were significantly higher in both the SS and SX groups of the total cohort in comparison with the XX control group. The data are as follows: RF (SS group vs XX control group: OR = 7.0, *P *= 0.0059, and SX group vs XX control group: OR = 6.3, *P *= 0.0126) and aCCP (SS group vs XX control group: OR = 10.2, *P *= 0.0010, and SX group vs XX control group: OR = 9.2, *P *= 0.0028).

**Table 5:** Relationship between human leucocyte antigen-DRB1 shared epitope homozygosity or heterozygosity and risk of anticyclic citrullinated peptide antibody or rheumatoid factor seropositivity

	Total cohort (*n *= 143)				Blacks (*n *= 124)			
	SS (*n *= 80)		SX (*n *= 52)		SS (*n *= 72)		SX (*n *= 43)	
	OR (95% CI)	*P *value	OR (95% CI)	*P *value	OR (95% CI)	*P *value	OR (95% CI)	*P *value
RF	7.0 (1.8 to 26.6)	0.0059	6.3 (1.5 to 25.7)	0.0126	4.5 (1.0 to 19.5)	0.0537	4.1 (0.9 to 19.3)	0.0813
aCCP	10.2 (2.6 to 40.3)	0.0010	9.2 (2.2 to 38.9)	0.0028	7.1 (1.6 to 30.4)	0.0121	6.4 (1.4 to 30.1)	0.0223

aCCP = anticyclic citrullinated peptide antibodies; HLA = human leucocyte antigen; RF = rheumatoid factor; SE = shared epitope; SS = homozygous for shared epitope allele; SX = heterozygous for shared epitope allele.

The SS and SX groups of the African patients showed a statistically significant predisposition to aCCP seropositivity compared with the XX group (SS group vs XX control group: OR = 7.01, *P *= 0.0121; SX group vs XX control group: OR = 6.4, *P *= 0.0223), and RF was also more prevalent in the SS and SX groups compared to the XX control group, although the difference was not significant (Table 5). Stratifying the patients according to du Montcel *et al*.'s allele classification [7] as SS, SX or XX therefore seems to strengthen the genetic association with the presence of aCCP, an observation which is in agreement with the findings of Huizinga *et al*. [14] and Klareskog *et al*. [39].

Further analysis was undertaken to probe the associations with the amino acids at positions 70 and 71 (S1, S2, S3D and S3P) with aCCP seropositivity by comparing noncarriers with the carrier allele groups. As shown in Table 6, this analysis revealed significantly higher ORs in the case of the S2 and S3P allele groups and weak, albeit significant, associations with the S1, S3D and X allele groups. RF seropositivity was weakly associated with all allele groups.

**Table 6:** Association between anticyclic citrullinated peptide antibody and rheumatoid factor with shared epitope alleles according to the du Montcel classification

	aCCP		RF	
	
Allele	OR (95% CI)	*P *value	OR (95% CI)	*P *value
S1 allele (*n *= 55)	2.9 (0.7 to 12.4)	0.2054	4.5 (1.2 to 16.9)	*0.0291
S2 allele (*n *= 28)	12.0 (2.4 to 59.5)	0.0019**	8.3 (1.6 to 43.3)	*0.0122
S3D allele (*n *= 38)	7.5 (1.8 to 31.4)	0.0100*	5.3 (1.3 to 22.7)	*0.0246
S3P allele (*n *= 61)	13.3 (3.2 to 54.4)	0.0003***	4.5 (1.2 to 16.8)	*0.0264
X allele (*n *= 64)	4.7 (1.2 to 17.6)	0.0213*	2.7 (0.8 to 9.8)	0.1678

aCCP = anticyclic citrullinated peptide antibodies; RF = rheumatoid factor. du Montcel classification is from du Montcel *et al*. [7]. OR (95% CI) and *P *values were calculated using Fisher's exact test to compare with SE noncarrier rheumatoid arthritis patients (*n *= 12). *P < 0.05. **P ≤ 0.001 and ***P ≤ 0.0003 values are highly significant.

The association between SE genotype and aCCP or RF seropositivity was further probed by categorising patients into high-risk or low-risk allele groups according to the du Montcel classification system [7] with the XX genotype used as a comparator. The low-risk genotype groups were (1) S1, consisting of genotypes S1/S1 + S1/X; (2) S3D, consisting of S3D/S3D + S3D/X; and (3) S1/S3D, consisting of S1/S1 + S1/X + S3D/S3D + S3D/X + S1/S3D. The high-risk genotype groups were (1) S2, consisting of genotypes S2/S2 + S2/X; (2) S3P, consisting of S3P/S3P + S3P/X; and (3) S2/S3P, consisting of S2/S2 + S2/X + S3P/S3P + S3P/X + S2/S3P. As shown in Table 7, these comparisons revealed a highly significant risk associated with the S2/S3P, S3P and S1/S3D groups and, to a lesser extent, S1 and S3D as described in previous studies [21,40]. No predisposing effect associated with RF seropositivity was identified, which confirms that the SE alleles are primarily associated with aCCP.

**Table 7:** Predisposing effect of shared epitope genotypes on anticyclic citrullinated peptide antibody and rheumatoid factor seropositivity

	aCCP		RF	
	
SE groups	OR (95% CI)	*P *value	OR (95% CI)	*P *value
S1 (*n *= 24)	2.4 (1.1 to 5.4)	0.0113*	1.6 (0.9 to 2.9)	0.1245
S3D (*n *= 17)	2.3 (1.0 to 5.4)	0.0287*	1.7 (0.9 to 3.0)	0.1059
S1/S3D (*n *= 50)	2.3 (1.0 to 5.3)	0.0049**	1.6 (0.9 to 2.9)	0.0611
S2 (*n *= 11)	2.2 (0.9 to 5.2)	0.0995	1.6 (0.9 to 3.1)	0.1930
S3P (*n *= 28)	2.5 (1.1 to 5.6)	0.0075**	1.6 (0.9 to 3.0)	0.0563
S2/S3P (*n *= 47)	2.4 (1.1 to 5.5)	0.0025**	1.6 (0.9 to 2.8)	0.0687

aCCP = anticyclic citrullinated peptide antibodies; RF = rheumatoid factor; SE = shared epitope. OR (95% CI) and *P *values were calculated using Fisher's exact test to compare rheumatoid arthritis patients with RA patients with the X/X genotype (*n *= 12). *P < 0.05. **P ≤ 0.008 values are highly significant. Groups S1, S3D, S2 and S3P consisted of the pooled homozygotes (SS) and heterozygotes (SX) for each group. Group S1/S3D consisted of S1/S1, S1/X, S3D/S3D, S3D/X and S1/S3D. Group S2/S3P consisted of S2/S2, S2/X, S3P/S3P, S3P/X and S2/S3P.

### Relationship of shared epitope with other circulating biomarkers and clinical markers of disease activity

Analysis (one-way ANOVA and Mann-Whitney *U *test) of the median values of the other circulating biomarkers and clinical markers of disease activity revealed significant associations with CRP (SS vs SX vs XX: *P *= 0.0041; SS vs SX: *P *= 0.0018), SAA (SS vs SX vs XX: *P *= 0.0018; SS vs SX: *P *= 0.0005), TNF (SS vs SX vs XX: *P *= 0.0295), IL-1Ra (SS vs XX: *P *= 0.0311) and DAS28 (SS vs SX vs XX: *P *= 0.0324; SS vs XX: *P *= 0.0103; SX vs XX: *P *= 0.0210). These trends were also noted with regard to most of the other cytokines, although they were not statistically significant (IL-1β, IL-2, IL-4, IL-6, IL-8, IL-12, CCL4, G-CSF, GM-GSF and IFN-γ).

Statistical comparison of the S1, S2, S3D and S3P groups did not reveal any significant differences, but in general the highest means for acute phase reactants, proinflammatory or anti-inflammatory cytokines, chemokines and VEGF were located predominantly in the SS-associated groups (data not shown).

## Discussion

Relatively few studies have been undertaken to evaluate the occurrence of RA-associated HLA-DRB1 alleles in southern African people with RA. The results of the current study, on the basis of high-resolution typing procedures in combination with the du Montcel HLA-DRB1 SE classification system [7], reveal that the incidence of RA risk-associated alleles in a population of predominantly black South African females with RA is comparable to or higher than those reported in European and Japanese populations [19-21] and somewhat higher than those reported in the relatively few studies undertaken in African Americans, which range from 25% to 40% [35-37]. Our data are in agreement with those of an earlier study where low-resolution PCR typing procedures were used, in which HLA-DRB1*04 conferred the most significant risk of RA in a cohort of black South African RA patients [38]. According to the du Montcel classification system [7], the highest risk of RA is associated with the S2 and S3P alleles. Recently, Barnetche *et al*. [19] reported an association between RA susceptibility and HLA-DRB1 alleles (categorized according to the du Montcel classification system [7]) in a combined analysis of worldwide samples (1,210 cases of RA), which included 23 San people of southern African origin. Although the number of cases was small, 52.2% of the patients were found to be carriers of the S2 RA susceptibility allele compared to 26% in the control group (OR = 3.05) [19]. In addition, in agreement with previous European and Japanese RA cohort studies [19,40], we observed highly significant associations of SS and SX with seropositivity for aCCP but not RF [21,40]. We have previously reported that isolated measurement of aCCP is no more accurate than RF in the serodiagnosis of RA [41]. However, when used in combination with SE genotyping, measurement of aCCP appears to distinguish a subset of patients who may differ with respect to response to therapy and outcome.

These observations appear to support the relationship between HLA-DRB1 SE subtypes, presentation of citrullinated epitopes and development of RA [42]. This contention is underscored by the broad associations of circulating cytokines with the du Montcel classification system risk phenotype [7]. SS is most strongly associated with T-helper cell 1 (GM-CSF, TNF, IFN-γ and IL-2), Th2 (IL-4 and IL-6) and macrophage (IL-1β, IL-6, IL-8, IL-12, TNF and VEGF) cytokines, as well as with IL-1Ra. We and others have previously reported that RA is associated with a generalised increase in circulating proinflammatory and anti-inflammatory cytokines and chemokines, which might be compatible with Th1, macrophage and fibroblast activation and a counterregulatory role of Th2 cells [22,23,43].

Our findings are somewhat at variance with those recently reported by Singwe-Ngandeu *et al*. [44] in a relatively small group of Cameroonian RA patients (*n *= 56). Thirty percent of the RA patients in their study were either SS or SX compared to 10% in patients with other inflammatory rheumatic diseases and 14% in healthy controls. However, no significant associations between SE positivity (SS or SX) and aCCP or RF were detected. Several possible reasons might explain the differences between their study and our present study. Notwithstanding the relatively small number of patients, these differences include the number of alleles typed (21 in Singwe-Ngandeu *et al*. vs 43 in our present study) and, most importantly, the effects of chemotherapy. Whereas the patients in our study were corticosteroid- and DMARD-naïve at the time of presentation, the patients in the Singwe-Ngandeu *et al*. study were receiving prednisone (91%), methotrexate (77%), sulfasalazine (12%), azathioprine (5%), leflunomide (5%) and D-penicillamine (2%). Although this difference may explain the absence of associations of SE alleles with aCCP and RF, it is unlikely to explain the differences in frequencies of SE alleles detected in the two studies. Notwithstanding the larger number of alleles typed in the current study, it is also possible that our patients represent those at the extreme end of the severe disease spectrum. In the health care setting of the developing world, the presentation of patients to specialised RA clinics, of which there are few, is likely to be delayed, and our study cohort may therefore reflect a selection bias of patients seen in a tertiary setting.

## Conclusions

The results of the current study demonstrate an increased frequency of high-risk SE alleles in a predominantly black South African population with RA. We have also shown a clear association of high-risk SE alleles with aCCP in particular, to a lesser extent with RF, with circulating cytokines and chemokines and possibly with disease severity.

## Abbreviations

aCCP: anticyclic citrullinated peptide antibodies; CRP: C-reactive protein; DAS28: Disease Activity Score in 28 joints; DMARD: disease-modifying antirheumatic drug; HLA: human leucocyte antigen; IFN: interferon; IL: interleukin; mAb = monoclonal antibody; PCR: polymerase chain reaction; RA: rheumatoid arthritis; RF: rheumatoid factor; SE: shared epitope; SS: homozygous for shared epitope allele; SX: heterozygous for shared epitope allele; Th: T helper cell; TNF: tumour necrosis factor; XX: no shared epitope allele.

## Competing interests

The authors declare that they have no competing interests.

## Authors' contributions

PWAM was responsible for the study design and the generation, analysis and communication of data. BH and MA were responsible for the study design, clinical assessment, and analysis and communication of data. EM was responsible for the statistical design and data analysis. AAW was responsible for the study design and communication of data. HF performed laboratory investigations and data analysis. MT was responsible for the study design, served as the clinical coordinator, and performed data analysis and communication. RA was responsible for the study design and the analysis and communication of data. All authors read and approved the final manuscript.

## References

[B1] Pratt AG Isaacs JD Mattey DL Current concepts in the pathogenesis of early rheumatoid arthritis Best Pract Res Clin Rheumatol 2009 23 37 48 10.1016/j.berh.2008.08.002 19233044 PMC2652659

[B2] Nepom GT Seyfried CE Holbeck SL Wilske KR Nepom BS Identification of HLA-Dw14 genes in DR4+ rheumatoid arthritis Lancet 1986 2 1002 1005 2877172 10.1016/s0140-6736(86)92614-0

[B3] Winchester R Genetic determination of susceptibility and severity in rheumatoid arthritis Ann Intern Med 1992 117 869 871 1416564 10.7326/0003-4819-117-10-869

[B4] Winchester R The molecular basis of susceptibility to rheumatoid arthritis Adv Immunol 1994 56 389 466 7521116 10.1016/s0065-2776(08)60456-3

[B5] Gregersen PK Silver J Winchester RJ The shared epitope hypothesis: an approach to understanding the molecular genetics of susceptibility to rheumatoid arthritis Arthritis Rheum 1987 30 1205 1213 10.1002/art.1780301102 2446635

[B6] Gao XJ Brautbar C Gazit E Segal R Naparstek Y Livneh A Stastny P A variant of HLA-DR4 determines susceptibility to rheumatoid arthritis in a subset of Israeli Jews Arthritis Rheum 1991 34 547 551 10.1002/art.1780340506 2025308

[B7] du Montcel ST Michou L Petit-Teixeira E Osorio J Lemaire I Lasbleiz S Pierlot C Quillet P Bardin T Prum B Cornelis F Clerget-Darpoux F New classification of HLA-DRB1 alleles supports the shared epitope hypothesis of rheumatoid arthritis susceptibility Arthritis Rheum 2005 52 1063 1068 10.1002/art.20989 15818663

[B8] Michou L Croiseau P Petit-Teixeira E du Montcel ST Lemaire I Pierlot C Osorio J Frigui W Lasbleiz S Quillet P Bardin T Prum B Clerget-Darpoux F Cornélis F European Consortium on Rheumatoid Arthritis Families Validation of the reshaped shared epitope HLA-DRB1 classification in rheumatoid arthritis Arthritis Res Ther 2006 8 R79 10.1186/ar1949 16646982 PMC1526640

[B9] Schellekens GA de Jong BA van den Hoogen FH van de Putte LB van Venrooij WJ Citrulline is an essential constituent of antigenic determinants recognized by rheumatoid arthritis-specific autoantibodies J Clin Invest 1998 101 273 281 10.1172/JCI1316 9421490 PMC508564

[B10] Mattey DL Hassell AB Plant MJ Cheung NT Dawes PT Jones PW Thomson W Poulton KV Hajeer AH Ollier WE The influence of HLA-DRB1 alleles encoding the DERAA amino acid motif on radiological outcome in rheumatoid arthritis Rheumatology (Oxford) 1999 38 1221 1227 10.1093/rheumatology/38.12.1221 10587549

[B11] van Boekel MA Vossenaar ER van den Hoogen FH van Venrooij WJ Autoantibody systems in rheumatoid arthritis: specificity, sensitivity and diagnostic value Arthritis Res 2002 4 87 93 11879544 10.1186/ar395 PMC128920

[B12] Hill JA Southwood S Sette A Jevnikar AM Bell DA Cairns E Cutting edge: the conversion of arginine to citrulline allows for a high-affinity peptide interaction with the rheumatoid arthritis-associated HLA-DRB1*0401 MHC class II molecule J Immunol 2003 171 538 541 12847215 10.4049/jimmunol.171.2.538

[B13] Auger I Sebbag M Vincent C Balandraud N Guis S Nogueira L Svensson B Cantagrel A Serre G Roudier J Influence of HLA-DR genes on the production of rheumatoid arthritis-specific autoantibodies to citrullinated fibrinogen Arthritis Rheum 2005 52 3424 3432 10.1002/art.21391 16255019

[B14] Huizinga TW Amos CI van der Helm-van Mil AH Chen W van Gaalen FA Jawaheer D Schreuder GM Wener M Breedveld FC Ahmad N Lum RF de Vries RR Gregersen PK Toes RE Criswell LA Refining the complex rheumatoid arthritis phenotype based on specificity of the HLA-DRB1 shared epitope for antibodies to citrullinated proteins Arthritis Rheum 2005 52 3433 3438 10.1002/art.21385 16255021

[B15] Raza K Breese M Nightingale P Kumar K Potter T Carruthers DM Situnayake D Gordon C Buckley CD Salmon M Kitas GD Predictive value of antibodies to cyclic citrullinated peptide in patients with very early inflammatory arthritis J Rheumatol 2005 32 231 238 15693082 PMC3160476

[B16] Samanci N Ozdem S Akbas H Mutlu D Gultekin M Arman M Donmez L Diagnostic value and clinical significance of anti-CCP in patients with advanced rheumatoid arthritis J Natl Med Assoc 2005 97 1120 1126 16173327 PMC2575986

[B17] de Vries RRP Huizinga TWJ Toes REM HLA and RA revisited: citrullinated food for the SE hypothesis, the DR6 effect, and NIMA Hum Immunol 2006 67 454 459 10.1016/j.humimm.2006.03.016 16728269

[B18] Quinn MA Gough AKS Green MJ Devlin J Hensor EMA Greenstein A Fraser A Emery P Anti-CCP antibodies measured at disease onset help identify seronegative rheumatoid arthritis and predict radiological and functional outcome Rheumatology (Oxford) 2006 45 478 480 10.1093/rheumatology/kei203 16287917

[B19] Barnetche T Constantin A Cantagrel A Cambon-Thomsen A Gourraud P New classification of HLA-DRB1 alleles in rheumatoid arthritis susceptibility: a combined analysis of worldwide samples Arthritis Res Ther 2008 10 R26 10.1186/ar2379 18307784 PMC2374469

[B20] Okada Y Yamada R Suzuki A Kochi Y Shimane K Myouzen K Kubo M Nakamura Y Yamamoto K Contribution of a haplotype in the HLA region to anti-cyclic citrullinated peptide antibody positivity in rheumatoid arthritis, independently of HLA-DRB1 Arthritis Rheum 2009 60 3582 3590 10.1002/art.24939 19950296

[B21] Gyetvai A Szekanecz Z Soos L Szabo Z Fekete A Kapitany A Teodorescu M Sipka S Szegedi G Lakos G New classification of the shared epitope in rheumatoid arthritis: impact on the production of various anti-citrullinated protein antibodies Rheumatology (Oxford) 2010 49 25 33 10.1093/rheumatology/kep338 19920092

[B22] Kokkonen H Söderström I Rocklöv J Hallmans G Lejon K Rantapää Dahlqvist S Up-regulation of cytokines and chemokines predates the onset of rheumatoid arthritis Arthritis Rheum 2010 62 383 391 20112361 10.1002/art.27186

[B23] Deane KD O'Donnell CI Hueber W Majka DS Lazar AA Derber LA Gilliland WR Edison JD Norris JM Robinson WH Holers VM The number of elevated cytokines and chemokines in preclinical seropositive rheumatoid arthritis predicts time to diagnosis in an age-dependent manner Arthritis Rheum 2010 62 3161 3172 10.1002/art.27638 20597112 PMC2980824

[B24] Hitchon CA Alex P Erdile LB Frank MB Dozmorov I Tang Y Wong K Centola M El-Gabalawy HS A distinct multicytokine profile is associated with anti-cyclical citrullinated peptide antibodies in patients with early untreated inflammatory arthritis J Rheumatol 2004 31 2336 2346 15570632

[B25] Alex P Szodoray P Knowlton N Dozmorov IM Turner M Frank MB Arthur RE Willis L Flinn D Hynd RF Carson C Kumar A El-Gabalawy HS Centola M Multiplex serum cytokine monitoring as a prognostic tool in rheumatoid arthritis Clin Exp Rheumatol 2007 25 584 592 17888215

[B26] Chen DY Hsieh TY Chen YM Hsieh CW Lan JL Lin FJ Proinflammatory cytokine profiles of patients with elderly-onset rheumatoid arthritis: a comparison with younger-onset disease Gerontology 2009 55 250 258 10.1159/000164393 18849599

[B27] Boissier MC Cell and cytokine imbalances in rheumatoid synovitis Joint Bone Spine 2011 78 230 234 10.1016/j.jbspin.2010.08.017 20961791

[B28] Arnett FC Edworthy SM Bloch DA McShane DJ Fries JF Cooper NS Healey LA Kaplan SR Liang MH Luthra HS Medsger TA Jr Mitchell DM Neustadt DH Pinals RS Schaller JG Sharp JT Wilder RL Hunder GG The American Rheumatism Association 1987 revised criteria for the classification of rheumatoid arthritis Arthritis Rheum 1988 31 315 324 10.1002/art.1780310302 3358796

[B29] Inoue E Yamanaka H Hara M Tomatsu T Kamatani N Comparison of Disease Activity Score (DAS)28- erythrocyte sedimentation rate and DAS28- C-reactive protein threshold values Ann Rheum Dis 2007 66 407 409 10.1136/ard.2006.054205 16926186 PMC1856019

[B30] Wells GA Patient-driven outcomes in rheumatoid arthritis J Rheumatol 2009 36 33 38 10.3899/jrheum.090129 19509328

[B31] Robinson J Waller MJ Parham P De Groot N Bontrop R Kennedy LJ Stoehr P Marsh SGE IMGT/HLA and IMGT/MHC: sequence databases for the study of the major histocompatibility complex Nucleic Acids Res 2003 31 311 314 10.1093/nar/gkg070 12520010 PMC165517

[B32] Robinson J Waller MJ Fail SC McWilliam H Lopez R Parham P Marsh SGE The IMGT/HLA database Nucleic Acids Res 2009 37 D1013 D1017 10.1093/nar/gkn662 18838392 PMC2686596

[B33] Middleton D Menchaca L Rood H Komerofsky R New allele frequency database Tissue Antigens 2003 61 403 407 http://www.allelefrequencies.net 10.1034/j.1399-0039.2003.00062.x 12753660

[B34] Mbayo K Mbuyi-Muamba JM Hallé L Salmon D Martageix C Castellano F Kaplan C Lurhuma Z HLA-DR gene frequencies in a Zairean population with particular reference to rheumatic diseases Clin Rheumatol 1998 17 105 109 10.1007/BF01452254 9641505

[B35] Del Rincόn I Battafarano DF Arroyo RA Murphy FT Fisbach M Escalante A Ethnic variation in the clinical manifestations of rheumatoid arthritis: role of HLA-DRB1 alleles Arthritis Care Res 2003 49 200 208 10.1002/art.11000 12687511

[B36] Hughes LB Morrison D Kelley JM Padilla MA Vaughan LK Westfall OA Dwivedi H Mikuls TR Holers VM Parrish LA Alarcόn GS Jonas BL Callahan LF Smith EA Gilkeson GS Howard G Moreland LW Patterson N Reich D Bridges SL Jr The HLA-DRB1 shared epitope is associated with susceptibility to rheumatoid arthritis in African Americans through European genetic admixture Arthritis Rheum 2008 58 349 358 10.1002/art.23166 18240241 PMC3726059

[B37] Mikuls TR Sayles H Yu F Levan T Gould KA Thiele GM Conn DL Jonas BL Callahan LF Smith E Brasington R Moreland LW Reynolds RJ Bridges SL Jr Associations of cigarette smoking with rheumatoid arthritis in African Americans Arthritis Rheum 2010 62 3560 3568 10.1002/art.27716 20722010 PMC2995845

[B38] Meyer PWA Brighton SW Anderson R Association of the HLA-DRB1*04 allele and its subtypes with rheumatoid arthritis in South Africa S Afr J Sci 2004 100 305 306

[B39] Klareskog L Rönnelid J Lundberg K Padyukov L Alfredsson L Immunity to citrullinated proteins in rheumatoid arthritis Ann Rev Immunol 2008 26 206 209 10.1146/annurev.immunol.26.021607.090244 18173373

[B40] Engelmann R Eggert M Neeck G Mueller-Hilke B The impact of HLA-DRB alleles on the subclass titres of antibodies against citrullinated peptides Rheumatology (Oxford) 2010 49 1862 1866 10.1093/rheumatology/keq179 20584722

[B41] Hodkinson B Meyer PW Musenge E Ally MM Wadee AA Anderson R Tikly M The diagnostic utility of the anti-CCP antibody test is no better than rheumatoid factor in South Africans with early rheumatoid arthritis Clin Rheumatol 2010 29 615 618 10.1007/s10067-010-1374-x 20127131

[B42] Szodoray P Szabó Z Kapitány A Gyetvai Á Lakos G Szántó S Szücs G Szekanecz Z Anti-citrullinated protein/peptide autoantibodies in association with genetic and environmental factors as indicators of disease outcome in rheumatoid arthritis Autoimmun Rev 2010 9 140 143 10.1016/j.autrev.2009.04.006 19427413

[B43] Meyer PWA Hodkinson B Mahmood A Musenge E Wadee AA Fickl H Tikly M Anderson R Circulating cytokine profiles and their relationship with autoantibodies, acute phase reactants, and disease activity in patients with rheumatoid arthritis Mediators Inflamm 2010 2010 158154 10.1155/2010/158514 PMC3061216 21437211

[B44] Singwe-Ngandeu M Finckh A Bas S Tiercy JM Gabay C Diagnostic value of anti-cyclic citrullinated peptides and association with HLA-DRB1 shared epitope alleles in African rheumatoid arthritis patients Arthritis Res Ther 2010 12 R36 10.1186/ar2945 20196860 PMC2888183

